# Phenylephrine, a common cold remedy active ingredient, suppresses uterine contractions through cAMP signalling

**DOI:** 10.1038/s41598-018-30094-5

**Published:** 2018-08-03

**Authors:** Xingjuan Chen, Marya Meroueh, Gabriela Mazur, Evan Rouse, Karmjot Singh Hundal, Christopher W. Stamatkin, Alexander G. Obukhov

**Affiliations:** 1 Department of Cellular and Integrative Physiology, Indiana University School of Medicine – Indianapolis, Indianapolis, Indiana 46202 USA; 2 Present Address: Advanced Testing Laboratory, Cincinnati, OH USA; 3 Present Address: Covance Greenfield Laboratories, Greenfield, IN USA

## Abstract

Regulation of uterine contractility is an important aspect of women’s health. Phenylephrine, a selective agonist of the α_1_-adrenoceptor and a potent smooth muscle constrictor, is widely used in women even during pregnancy to relieve cold-related symptoms, to treat postpartum haemorrhoid, and during routine eye exams. We performed isometric tension recordings to investigate the effect of phenylephrine on mouse uterine contractility. Phenylephrine decreased spontaneous and oxytocin-induced contractions in non-pregnant mouse uterine rings and strips with an IC_50_ of ~1 μM. Prazosin, an inhibitor of α_1_-adrenoceptor, did not prevent phenylephrine-mediated relaxations. Conversely, ICI118551, an antagonist of β2-adrenoceptors, inhibited phenylephrine relaxation. In the presence of ICI118551, high concentrations (>30 μM) of phenylephrine caused mouse uterine contractions, suggesting that β-adrenoceptor-mediated inhibition interferes with the phenylephrine contractile potential. Phenylephrine-dependent relaxation was reduced in the uterus of pregnant mice. We used primary mouse and human uterine smooth muscle cells (M/HUSMC) to establish the underlying mechanisms. Phenylephrine stimulated large increases in intracellular cAMP in M/HUSMCs. These cAMP transients were decreased when HUSMCs were cultured in the presence of oestrogen and progesterone to mimic the pregnancy milieu. Thus, phenylephrine is a strong relaxant in the non-pregnant mouse uterus, but exhibits diminished effect in the pregnant uterus.

## Introduction

Excessive uterine contractility is strongly associated with primary dysmenorrhea (menstrual pain/cramps) in young women^1,2^ and preterm labour in pregnant women^3–6^. Primary dysmenorrhea requiring medications to reduce pain occurs in up to 55% of young women^7^; and preterm labour complicates up to 10% of pregnancies^8^. Over-the-counter cold-flu medications are extensively used in women, particular phenylephrine formulations. Although phenylephrine is a potent smooth muscle constrictor, little work has been done to explore its effect on uterine contractility.

Phenylephrine is clinically used to treat hypotension as a vasoconstrictor. Phenylephrine is also one of the most widely consumed over-the-counter remedies with a broad range of therapeutic uses. Indicated for nasal decongestion, phenylephrine is a key active ingredient in several cold-relieving formulations, such as Vicks Dayquil Cold & Flu Relief, Theraflu Multi-Symptom Severe Cold, Robitussin Peak Cold Daytime Cold + Flu and others, which are taken by millions of pregnant and non-pregnant women. The nasal decongesting effect is attributed to phenylephrine’s potential of constricting vascular smooth muscle. Topical phenylephrine is widely used to treat postpartum haemorrhoids in women^9,10^. Its ophthalmic formulation is used to constrict the iris dilator smooth muscles, which causes pupil dilation allowing visualization of the retina during routine eye exams. It has been demonstrated that intranasal, ophthalmic, or oral administration of phenylephrine may result in its systemic concentration surge within the micromolar range^11,12^.

Phenylephrine is an agonist of the α_1_-adrenergic receptor, which is believed to have practically no β-adrenergic activity^13^. The α_1_-adrenergic receptor is a G_q/11_ protein-coupled receptor. Upon activation of the α_1_-adrenergic receptor, the heterotrimeric G protein, G_q/11_, dissociates to release α and βγ subunits, which in turn activate phospholipase C (PLC) inducing the phosphoinositide signalling cascade. PLC activation leads to an increase in intracellular IP_3_ that binds to the IP_3_ receptor on the endoplasmic reticulum to cause calcium release from intracellular stores, followed by calcium influx, eventually triggering smooth muscle contraction.

Uterine smooth muscles express both α and β adrenoceptors^14–17^. α adrenoceptor activation increases uterine contractility^15,16^. Conversely, agonists of β2 adrenoceptors cause a potent relaxation and are clinically used to prevent preterm labor^18^. The expression level of adrenoceptors is altered in the pregnant uterus^15,16^.

We performed isometric tension measurements in mouse uterine rings to investigate the effects of phenylephrine on uterine contractility. Primary uterine smooth muscle cells were used to investigate the molecular mechanisms underlying the compound action. Surprisingly, our data revealed that phenylephrine has a potent relaxant effect in the non-pregnant uterus at clinically-relevant concentrations.

## Results

### Phenylephrine inhibits spontaneous and oxytocin-induced contractions of the mouse uterus

**Figure 1:**
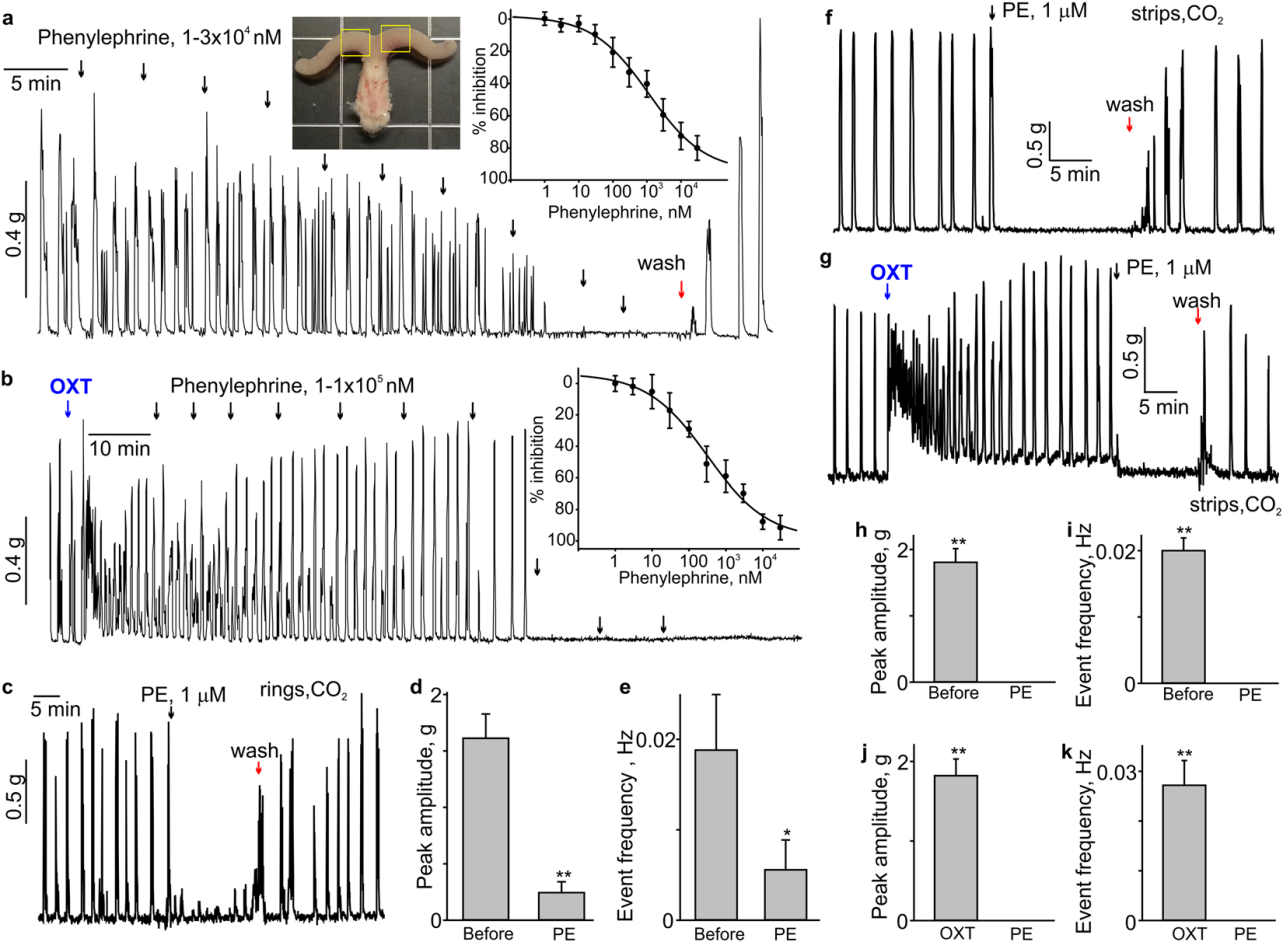
Phenylephrine inhibits the spontaneous and oxytocin-induced contractions in mouse uterus. (**a**) A sample trace shows the effect of phenylephrine (PE) on spontaneous contractility in a mouse uterine ring. The left inset shows a photograph of isolated mouse uterine horns and vagina. Yellow rectangles show the two segments of uterine horns typically used for isometric tension experiments. The dose-response curve for PE-induced relaxation is shown in the right inset (N = 8, the data were presented as mean ± S.E.). (**b**) A sample trace depicts that PE inhibits oxytocin-induced contractions in the mouse uterine ring in a concentration-dependent manner. The corresponding dose-response curve is shown in the inset (N = 6). (**c**) A sample trace shows the effect of 1 μM phenylephrine (PE) on a uterine ring isolated from CO_2_-anesthesized female mouse (N = 4). (**d**,**e**) Summary data for the frequency and amplitude of spontaneous contractions in uterine rings isolated from CO_2_-anesthesized female mice (N = 4). (**f**–**k**) Shown are sample traces and summary data for the effect of 1 μM phenylephrine (PE) on the frequency and amplitude of spontaneous and oxytocin (100 nM)-induced contractility in uterine strips from CO_2_-anesthesized female mice (N = 4). Active tension changes are shown in (**a**–**c**,**f**,**g**). The data were presented as means ± S.E. The value of “N” is the number of mice. The vertical arrows show the times where the drugs were added to the bath. OXT = oxytocin (100 nM).

Phenylephrine is a known smooth muscle constrictor and is clinically used to treat hypotension^19^. We first employed the isometric tension measurement approach to test whether phenylephrine increases the contractility of the non-pregnant mouse uterus. The uterine rings exhibited regular, spontaneous activity when 0.1–0.5 g preload was given (Fig. 1a). Unexpectedly, phenylephrine significantly decreased the frequency and amplitude of contractions when it was used at a concentration between 0.01–10 µM in mouse uterine rings with spontaneous activity (Fig. 1a). The right inset in Fig. 1a shows the concentration-dependent inhibition curve for phenylephrine. The apparent EC_50_ value for phenylephrine was 1.1 ± 0.7 µM (N = 8). As shown in Fig. 1b, 100 nM oxytocin, which is clinically used to induce labour, potently stimulated the uterus contractions. Phenylephrine dose-dependently inhibited oxytocin-induced contractions with an apparent EC_50_ value of 0.3 ± 0.2 µM (N = 6). In these experiments, we used isoflurane anaesthesia when mice were euthanized. To ensure that isoflurane anaesthesia is not responsible for the lack of phenylephrine-induced contractions, we investigated whether phenylephrine would constrict uterine rings and strips obtained from the mice euthanized under CO_2_ inhalation anaesthesia. We found that phenylephrine still caused potent relaxation in the uterine rings and strips obtained from mice, which were never exposed to isoflurane (Fig. 1c,f,g). Bar graphs in Fig. 1d,e,h–k show that phenylephrine significantly decreased both the peak amplitude and frequency of uterine contractions in both rings and strips, representing the activity of circular and longitudinal smooth muscle fibres, respectively.

### Phenylephrine inhibits uterine contractions by activating the β2-adrenoceptor

**Figure 2:**
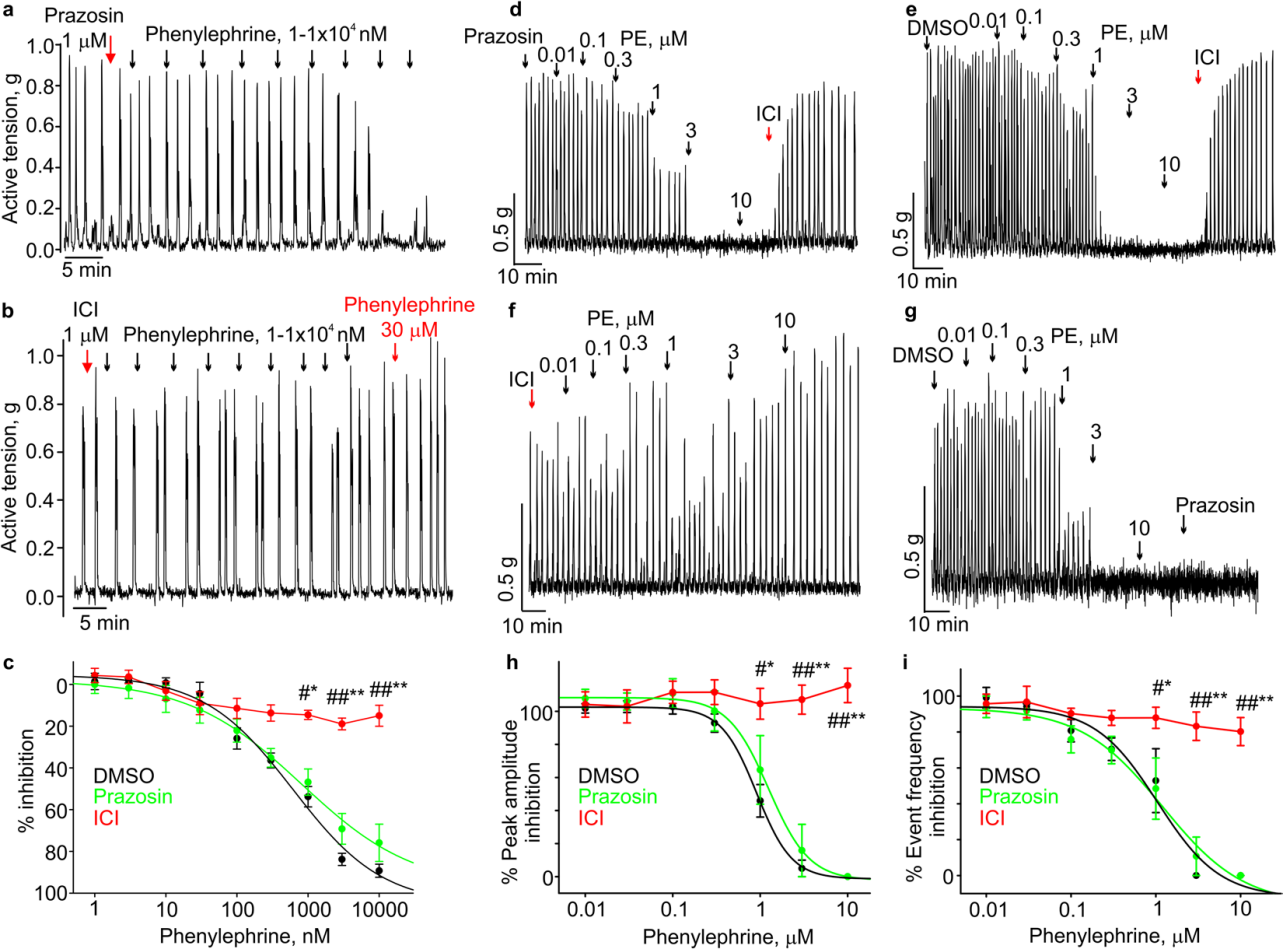
β2-adrenoceptor antagonist prevents the PE induced relaxation of spontaneous uterine contractions. (**a**) A sample trace shows that prazosin has no effect on PE-induced inhibition of spontaneous contractility in a mouse uterine ring. (**b**) A sample trace shows that ICI118551, a β2-adrenoceptor antagonist, prevents the inhibition induced by PE in a mouse uterine ring. **(c)** Summary for the date shown in **(a,b**). DMSO (N = 5) *vs* ICI118551 (N = 5), *p < 0.05, **p < 0.01; ICI118,551 *vs* Prazosin (N = 5), #p < 0.05, ##p < 0.01. (**d**–**g**) The sample traces show that ICI118551 prevents the inhibition induced by PE in the mouse uterine strips, whereas prazosin has no effect on the PE-induced inhibition of the spontaneous contractility’s frequency and amplitude. (**h**,**i**) Summary for the data shown in (**d**–**g**). DMSO (N = 4) *vs* ICI118551 (N = 4), *p < 0.05, **p < 0.01; ICI118551 *vs* Prazosin (N = 4), #p < 0.05, ##p < 0.01. The data were presented as means ± S.E. The value of “N” is the number of mice. Active tension changes are shown in **(a**,**b** and **d**–**g)**.

**Figure 3:**
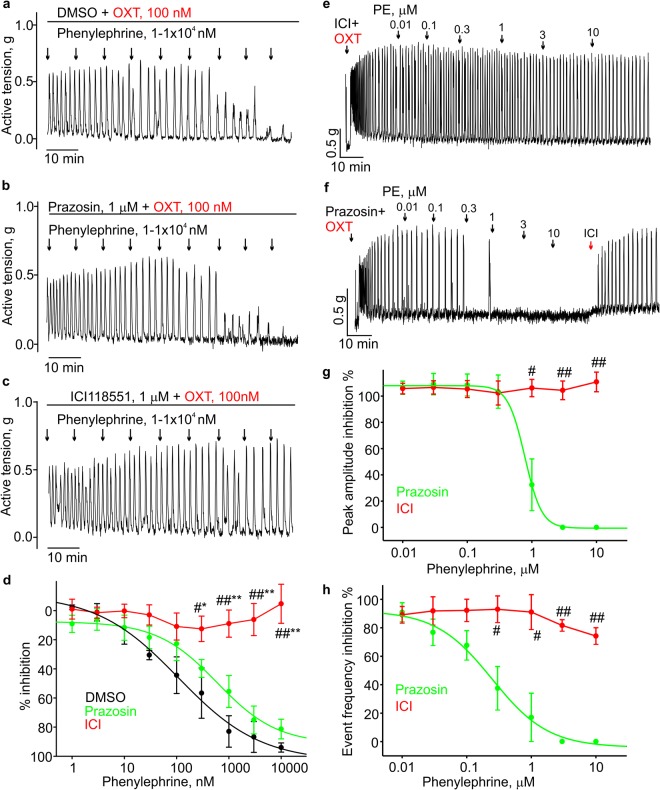
β2-adrenoceptor antagonist prevented the PE-induced relaxation of the uterus in presence of oxytocin. (**a**–**c**) Sample traces show that ICI118551, a β2-adrenoceptor antagonist, prevents the inhibition induced by PE in the mouse uterine rings, whereas prazosin has no effect on the PE-induced inhibition of oxytocin-induced contractions. (**d**) Concentration-inhibition relationships for the data shown in (**a**,**b**). DMSO (N = 3) vs ICI118551 (n = 8), *p < 0.05, **p < 0.01; ICI118551 vs Prazosin (N = 8), #p < 0.05, ##p < 0.01. (**e**,**f**) Sample traces show that ICI118551 prevents the inhibition induced by PE in the mouse uterine strips, whereas prazosin has no effect on the PE-induced inhibition of oxytocin-induced contractions’ frequency and amplitude. (**g**,**h**) Concentration-inhibition relationships for the data shown in **(e**,**f)**. ICI118551 (N = 4) *vs* Prazosin (N = 4), #p < 0.05, ##p < 0.01. The data were presented as means ± S.E. The value of “N” is the number of mice. Active tension changes are shown in (**a–c**,**e** and **f**).

After confirming that phenylephrine is a potent relaxant in the mouse uterus, we set out to investigate the mechanism of phenylephrine’s relaxant effect. Phenylephrine is a known agonist of the α1-adrenergic receptor. Therefore, we first tested whether its relaxant effect depends on the activation of the α1-adrenergic receptor by using prazozin, an antagonist of the α1-adrenergic receptor. 1 µM prazosin did not abolish the phenylephrine-induced relaxation of non-pregnant mouse uterine rings and strips in the presence (Fig. 2a,c,d,h and i) or absence of oxytocin (Fig. 3b,d and f). We next tested ICI118551, a β2-adrenergic receptor antagonist^20^. Pretreatment with 1 µM ICI118551 prevented the phenylephrine-induced inhibition in both spontaneously contracting uterine rings and strips (Fig. 2b,c,f,h and i) and oxytocin-stimulated uterine rings and strips (Fig. 3c–e). The data in Fig. 2c (N = 5), 2h (N = 4), and 2i (N = 4) and Fig. 3d (N = 8) showed that 1 µM ICI118551 significantly blocked the 1, 3, and 10 µM phenylephrine-induced relaxations, compared to the vehicle control (DMSO). DMSO did not affect phenylephrine-induced relaxations (Fig. 2c,e,g,h and i). Comparison of prazosin and ICI118551 effects on phenylephrine-induced relaxations in oxytocin-stimulated uterine strips is shown in Fig. 3g,h (N = 4). Figure 2d,e (N = 4) and Fig. 3f (N = 4) show that acute application of 1 µM ICI118551 potently reversed the 10 µM phenylephrine-induced relaxation in uterine strips. We noticed that phenylephrine increased uterine contractility at much higher concentrations (>30 µM) in the presence of 1 µM ICI118551 (Fig. 2b) likely due to its activation of the α_1_-adrenergic receptor. Thus, we concluded that relaxant effect of phenylephrine is mediated via activation of the β2 adrenergic receptor on uterine smooth muscle cells.

### Phenylephrine increased the intracellular cAMP levels in isolated mouse uterine smooth muscle cells

**Figure 4:**
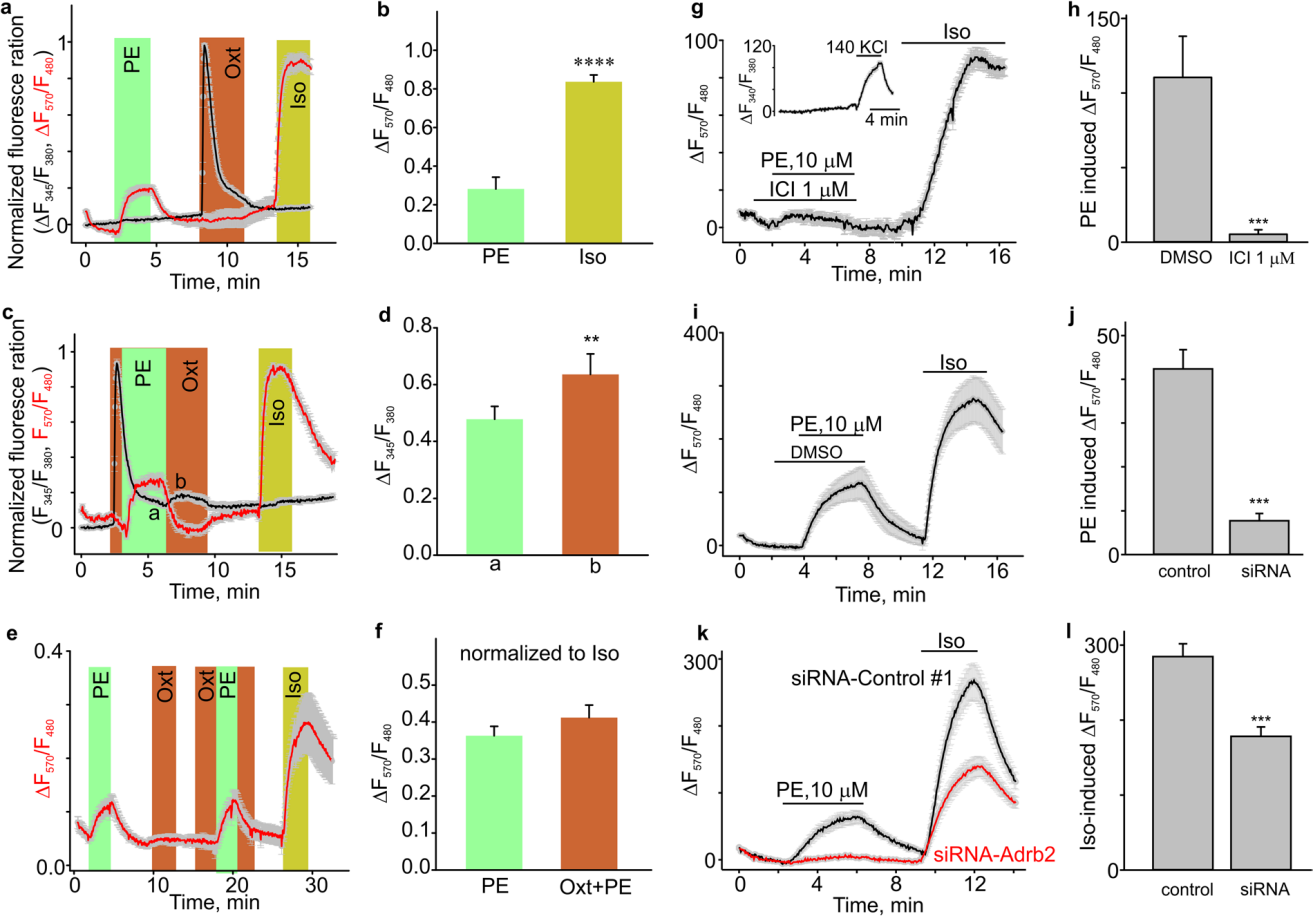
Phenylephrine stimulated the intracellular increase of cAMP levels in isolated mouse uterus smooth muscle cells. **(a**–**d)** The time courses show the changes of Fura-2 fluorescence ratio (F_345_/F_380_, black line) and cAMP reporter fluorescence intensity ratio (F_570_/F_480_, red line) on isolated mouse uterus smooth muscle cells transduced with the cAMP reporter construct. (**b**,**d**) Show the summary data from (**a**, n = 136) and (**b**, n = 216**)**. The fluorescence values were normalized to the baseline fluorescence level. To detect intracellular cAMP change, the Red Upward cADDis cAMP sensor was excited at 570 nm, whereas Green Downward cADDis cAMP sensor fluorescence was excited at 480 nm. Bars indicate the times when the applications of compounds took place. The data set is obtained from three to six repeats of independent experiments performed on different days. (**e**) The time course shows the cAMP reporter fluorescence intensity ratio (red line) changes in isolated mouse uterus smooth muscle cells infected with the cAMP reporter construct. **(f**) Summary for the data shown in (**e**, n = 28**)**. The data set is obtained from four independent experiments. **(g–i)** The time courses and summary data show that ICI118551 (n = 77), but not the vehicle DMSO (n = 51) prevents the PE-induced changes of cAMP reporter fluorescence intensity ratio (F_570_/F_480_) in isolated mouse uterine smooth muscle cells transduced with the cAMP reporter construct. The inset in (**g**) shows the time course of KCl-induced ΔF340/F380 ratio changes (black line). **(j**–**l)** The siRNA against the β2-adrenoceptor, but not the Negative Control #1 siRNA, prevented phenylephrine-induced cAMP rises in mouse uterine smooth muscle cells. (**k**) Shows the time courses for PE- and Iso-induced changes in cAMP levels in mouse uterine smooth muscle cells electroporated with the indicated siRNAs. PE failed to stimulate cAMP rises in mouse uterine smooth muscle cells electroporated with siRNA against the β2-adrenoceptor (n = 101), but induced cAMP rises in mouse uterine smooth muscle cells electroporated with Negative Control #1 siRNA (n = 91). PE, phenylephrine; Oxt, oxytocin; Iso, isoproterenol. **p < 0.01, ****p < 0.0001. The data were presented as means ± S.E. The grey vertical lines represent S.E. The “n” values indicate the number of cells, and each data set is obtained from three experiments.

The results of isometric tension experiments suggested that phenylephrine inhibited uterine contractions through activation of the β2 adrenergic receptor. Activation of the β adrenergic receptor leads to G_S_-heterotrimeric protein dissociation and consequent stimulation of adenylyl cyclase that produces 3′,5′-cyclic adenosine monophosphate (cAMP). Therefore, we then tested if phenylephrine modulates intracellular cAMP levels in uterine smooth muscle cells. In these experiments, we utilized a fluorescent cAMP-reporter assay. The cAMP reporter construct was transduced into uterine smooth muscle cells using a baculovirus-based delivery system. Phenylephrine (10 µM) increased the cytoplasmic cAMP concentration (n = 136, Fig. 4a,b), although the amount of cAMP production was smaller than that induced by isoproterenol (Iso, 10 µM), a positive control. The application of oxytocin did not affect phenylephrine-induced intracellular cAMP increases (n = 28, Fig. 4e,f). Notably, phenylephrine not only increased cAMP levels, but the drug also slightly reduced oxytocin-induced calcium increases (n = 216, Fig. 4c,d).

We next texted the effect of ICI118551 (1 µM) on the phenylephrine induced cAMP transients in mouse uterine smooth muscle cells. As shown in Fig. 4g–i, phenylephrine (10 µM) failed to increase the intracellular cAMP in the ICI118551 (1 µM)-pretreated uterine smooth muscle cells (n = 77), whereas the cells pre-treated with DMSO (n = 51) had normal cAMP responses to phenylephrine (10 µM). We found that the effect of ICI118551 was not reversible even after the cells were washed for several minutes with the standard Krebs solution. Similar observations were made during the isometric tension experiments in mouse uterine rings and strips.

To further verify the role of β2 adrenergic receptor in mediating phenylephrine-induced cAMP rises in mouse uterine smooth muscle cells, we utilized the siRNA approach. The siRNA targeting the β2 adrenergic receptor (siRNA-Adrb2) or Negative control (scrambled) siRNA was electroporated into mouse uterine smooth muscle cells. We found that siRNA-Adrb2-electroporated cells (n = 101) had a significantly smaller responses to phenylephrine compared to the negative control #1 siRNA-electroporated cells (n = 91, Fig. 4k,i). These data indicate that β2-adrenoceptor-dependent increases in the intracellular cAMP underlies phenylephrine relaxant effects on spontaneous and oxytocin-induced contractions in the mouse uterus.

### Caffeine decreases spontaneous and oxytocin-induced contractions in the mouse uterus

**Figure 5:**
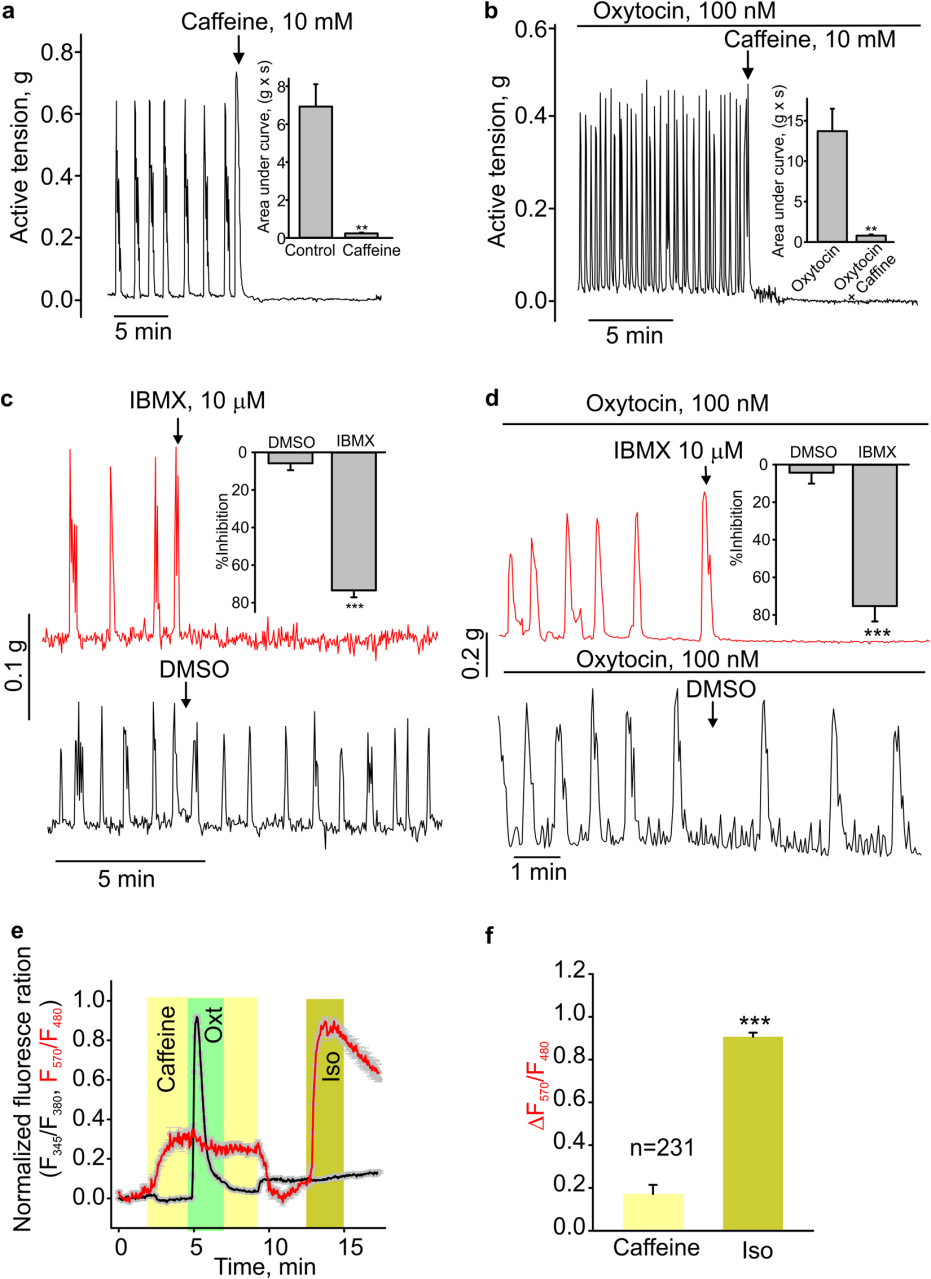
Caffeine decreased the uterine contractility by increasing the intracellular cAMP level. (**a**,**b**) The time courses of active tension changes are shown. Caffeine relaxed the mouse uterine rings in the absence (N = 5) or presence (N = 9) of oxytocin. The insets show the extent of caffeine-induced uterine relaxation. (**c**,**d**) The sample traces of active tension changes show that IBMX, but not DMSO relaxed the mouse uterus rings in absence (N = 6) or presence (N = 8) of oxytocin. The bar graphs demonstrate the extent of IBMX-induced uterine relaxation. (**e**,**f**) The time courses of fluorescence ratio changes. Furo-2 fluorescence changes (intracellular Ca^2+^ changes) are shown as the black line, and cAMP reporter fluorescence ratio changes are depicted as the red line. Bar graph (**f**) shows the summary data for E. Iso, isoproterenol. **p < 0.01, ***p < 0.001, n = 231. The data were presented as means ± S.E. The grey vertical lines represent S.E. The “n” values indicate the number of cells. Each data set is obtained from three to six repeats of independent experiments performed on different days. The value of “N” is the number of mice.

Caffeine was also reported to decrease the uterine contractility through inhibiting the activity of phosphodiesterase and then increasing cAMP^21^. We confirmed that 10 mM caffeine did increase intracellular cAMP levels (Fig. 5e,f) and significantly relaxed non-pregnant mouse uterine rings in the presence (N = 9) or absence (N = 5) of oxytocin (Fig. 5a,b). The application of IBMX, which inhibits phosphodiesterase reducing cAMP levels, also inhibited the spontaneous and oxytocin-stimulated contractility in uterine rings (Fig. 5c,d).

### Phenylephrine weakly inhibits the contractions in pregnant mouse uterine rings

**Figure 6:**
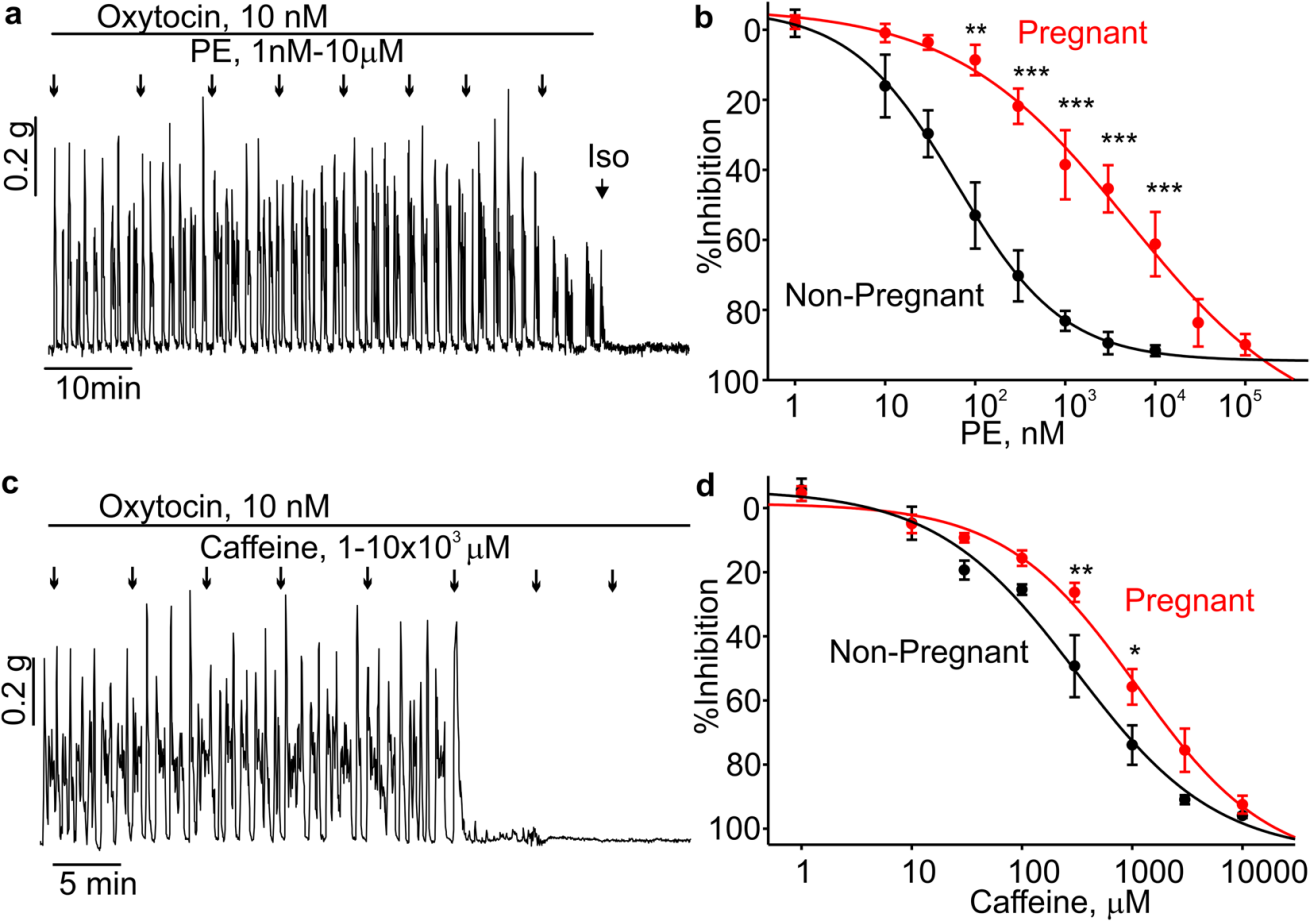
Phenylephrine-induced relaxation was less pronounced in pregnant mouse uterine rings. (**a**,**b**) A sample isometric tension trace and summary data are shown. Phenylephrine (PE) relaxed pregnant mouse uterine rings stimulated with oxytocin in a concentration-dependent manner (N = 5). (**c**,**d**) A sample trace and summary data show the caffeine-mediated inhibition of pregnant uterine ring contractions induced by oxytocin (N = 4). *p < 0.05, **p < 0.01, ***p < 0.001. The data were presented as means ± S.E. The value of “N” is the number of mice.

We next investigated whether phenylephrine caused relaxations of uterine rings from pregnant mice. Phenylephrine dose-dependently inhibited the uterine contractions at concentrations ranged from 0.1 to 10 µM. Percent inhibition for phenylephrine in pregnant uterus was significantly smaller at 0.1, 0.3, 1, 3 and 10 µM (Fig. 6a,b, N = 6) with an EC_50_ of 5.1 µM. We also tested the relaxation effects induced by caffeine in uterine rings from pregnant mice. As shown in Fig. 6c,d, the pregnant mouse uterine rings had less sensitivity to caffeine. Caffeine at 0.3 and 1.1 mM induced significantly smaller inhibition in pregnant mouse uterine rings (N = 4).

### The effect of phenylephrine on human uterine smooth muscle cells (HUSMCs)

**Figure 7:**
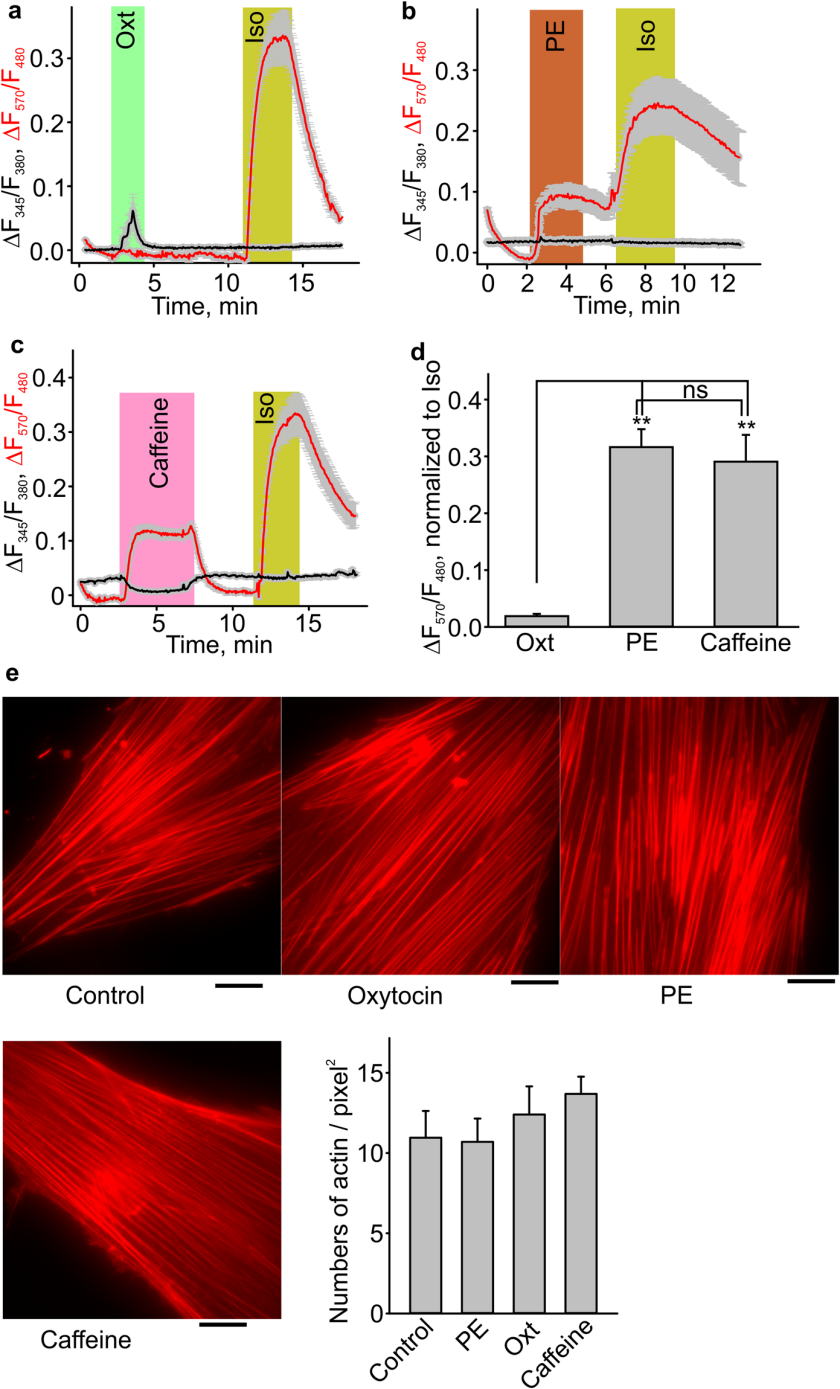
Phenylephrine (PE) and caffeine increased intracellular cAMP levels in HUSMCs. **(a**–**d)** Sample traces and summary data for phenylephrine (PE, n = 43), oxytocin (Oxt, n = 40), caffeine (n = 28) and isoproterenol (Iso)-induced cAMP reporter fluorescence ratio changes in HUSMCs. **(e)** F-actin stress fiber organization visualized with phalloidin-FITC in HUSMCs from control (n = 7), PE (n = 7), oxytocin (n = 6) and caffeine (n = 6) treated groups (scale bar indicates 20 µm). The right panel shows the summary data. **p < 0.01. The data were presented as means ± S.E. The grey vertical lines represent S.E. The “n” values indicate the number of cells. Each data set is obtained from three to five repeats of independent experiments performed on different days.

We also determined whether phenylephrine is affecting intracellular cAMP and calcium levels in primary HUSMCs that were transduced with the cAMP reporter construct. Oxytocin (100 nM) induced an increase in intracellular calcium but had no effect on cAMP levels in HUSMCs (Fig. 7a). Phenylephrine (10 µM, n = 43) and caffeine (10 mM, n = 40) increased intracellular cAMP levels in HUSMCs (Fig. 7b–d). Isoproterenol (10 µM) was used as a positive control in these experiments. We next investigated whether phenylephrine affects stress fibres in HUSMCs phalloidin-red, an f-actin marker. Figure 7e shows that the cytoskeletal organization in these treated groups was not affected in phenylephrine (n = 17), oxytocin (n = 16) or caffeine (n = 16) treated cells.

### The treatment with progesterone (P4) and estradiol-17β (E2) to mimic the pregnancy environment decreases the phenylephrine-induced cAMP levels

**Figure 8:**
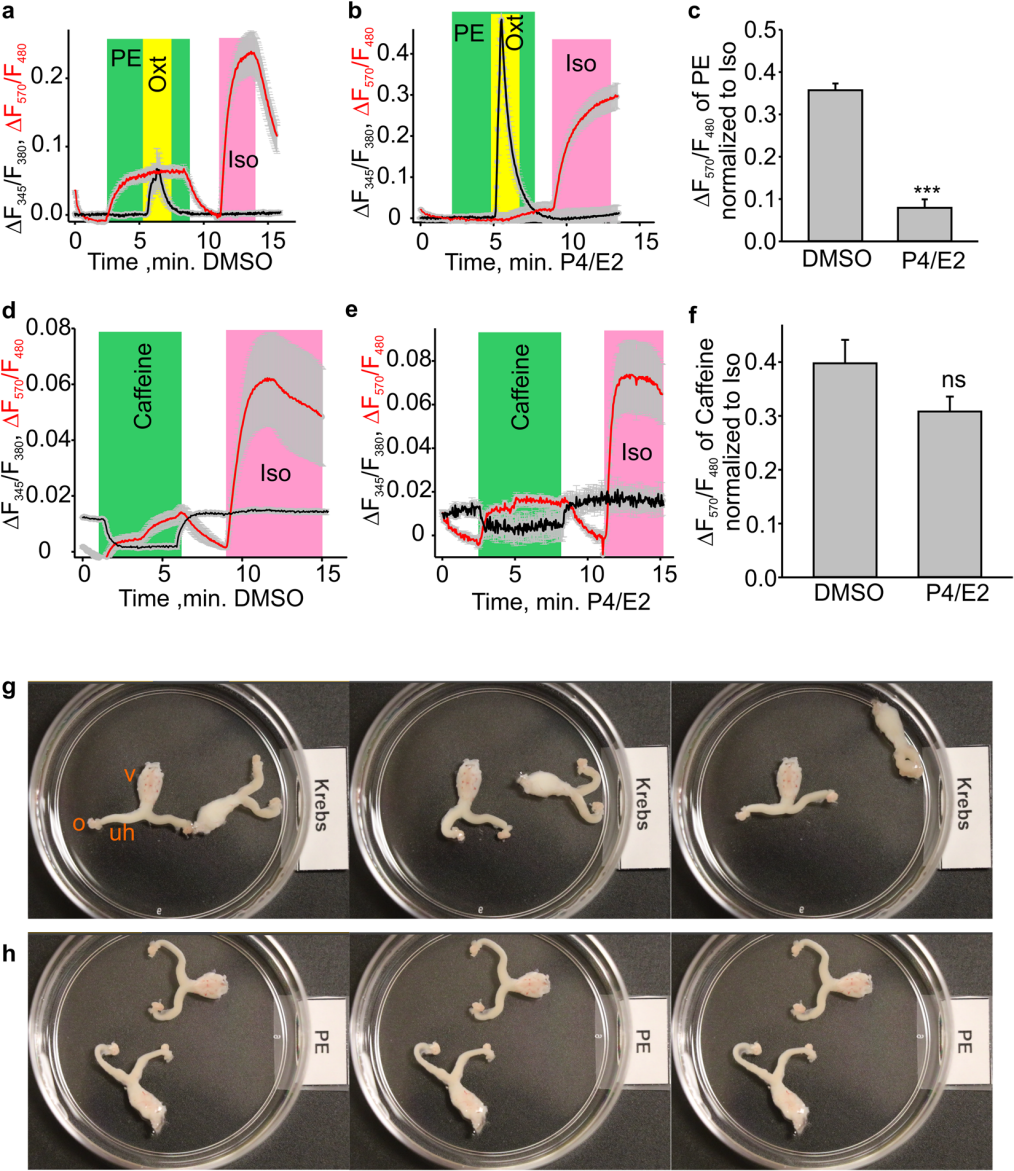
Effects of phenylephrine in HUSMCs and *ex vivo* female mouse reproductive tract preparations. (**a**–**c**) The time course of changes and bar graph show the phenylephrine (PE), oxytocin (Oxt) and isoproterenol (Iso)’s effects on the cAMP reporter and Fura-2 fluorescence ratios (DMSO, n = 95; P4/E2, n = 64). Phenylephrine induced smaller cAMP transients in the P4 and E2 treated HUSMCs. (**d**–**f**) The time course and bar graph show caffeine’s effect on the cAMP reporter and Fura-2 fluorescence ratios (DMSO, n = 38; P4/E2, n = 66). ***p < 0.001. The data were presented as means ± S.E. The grey vertical lines represent S.E. The “n” values indicate the number of cells. Each data set is obtained from three repeats of independent experiments performed on different days. (**g**,**h**) Show two *ex vivo* female mouse reproductive tract preparations. The sample images were consequently acquired with 10 second intervals in either the standard Krebs solution or in Krebs solution supplemented with 1 μM phenylephrine. The images were captured from the Supplementary video #1, which is available online. Phenylephrine markedly reduced spontaneous uterine horn peristalsis in the *ex vivo* female mouse reproductive tract preparations (N = 5). “o” = oviduct; “v” = vagina; “uh” = uterine horn.

Since phenylephrine was less effective in pregnant mouse uterine rings, we tested whether its effect on cAMP levels would be decreased in HUSMCs treated with P4 (1 µM) and E2 (100 nM) for 72 hours. We found that that phenylephrine indeed induced smaller cAMP rises in P4/E2-treated HUSMCs (n = 64) as compared to the vehicle control (DMSO)-treated HUSMCs (n = 95, Fig. 8a–c). On the other hand, the effect of caffeine on the intracellular cAMP was not different between the control and P4/E2 treatment groups. Thus, only the phenylephrine relaxant effect is dependent on the pregnancy status in the mouse uterus.

### Phenylephrine markedly decreased uterine peristalsis in *ex vivo* female mouse reproductive tract preparations

The uterus constantly exhibits spontaneous wavelike movements (uterine peristalsis). Therefore, we next set out to determine whether phenylephrine affects the spontaneous uterine peristalsis in an e*x vivo* female mouse reproductive tract preparation, which included the oviducts, uterine horns, uterine body, cervix, and vagina (Fig. 8g). We found that 1 μM phenylephrine potently decreased spontaneous uterine peristalsis (N = 5, Fig. 8h; see Supplementary video 1).

## Discussion

In this study, we found that phenylephrine potently decreased spontaneous and oxytocin induced contractions in the mouse uterus. Interestingly, the relaxant effect of phenylephrine was reduced in pregnant mouse uterus. We established that phenylephrine caused intracellular cAMP surges in MUSMCs due to the activation of the β2 adrenoreceptor, which was likely responsible for phenylephrine’s relaxant action. Phenylephrine-induced cAMP increases were also observed in primary HUSMCs, suggesting that phenylephrine may also exhibit tocolytic effects in the human uterus. Primary HUSMC cultures pretreated with a combination of P4 and E2 to mimic the pregnancy milieu showed reduced phenylephrine-induced cAMP surges, which is consistent with our mouse uterus data.

Phenylephrine is an over-the-counter medication, which is routinely used by millions of non-pregnant and pregnant women with few safety precautions. The phenylephrine blood plasma concentration may reach a peak concentration of 21 μM in a 70 kg healthy woman when a phenylephrine-containing over-the-counter formulation is consumed at recommended doses. Using a mouse uterus model, we established that phenylephrine has a significant relaxant effect at 1 μM. Although the relaxant effect was decreased in pregnant mice, 10 µM phenylephrine inhibited 50% of the oxytocin-induced contractility. Thus, the therapeutic doses of phenylephrine may modulate uterine contractility.

Exploring the underlying mechanisms of phenylephrine’s relaxant effect in the mouse uterus, we determined that phenylephrine acts via the β2-adrenergic receptor rather than the usual α1-adrenergic receptor pathway. The β-adrenergic receptor is coupled to the S-type of heterotrimeric G-proteins. Dissociation of G_S_ leads to an increased adenylyl cyclase activity and intracellular cAMP surges, which cause smooth muscle relaxation. To provide evidence for this mechanism, we used two antagonists, prazosin and ICI118551. Prazosin is an antagonist of the α1 receptor. ICI118551 is an antagonist of the β2 receptor. In uterine rings and strips pretreated with prazosin, phenylephrine was able to inhibit uterine contractions. Conversely, in uterine rings and strips pretreated with ICI118551, the inhibitory effect of phenylephrine was significantly diminished. Though both β2- and β3-adrenoceptors are expressed in mouse uterus^14,17^, the facts that ICI118551 completely prevented phenylephrine relaxations in our mouse uterine preparations and the siRNA against the β2-adrenoceptor was effective in decreasing phenylephrine-induced cAMP rises in isolated mouse uterine smooth muscle cells suggest that α-adrenoceptors and β3-adrenoceptors are unlikely to be involved. However, additional future studies utilizing β2-adrenoceptor knockout mice will be needed to determine whether the β3-adrenoceptor activation might contribute to phenylephrine-mediated relaxant action.

Interestingly, we noticed that phenylephrine increased uterine phasic contractions at the concentrations exceeding 30 μM in the presence of ICI118551 most likely due to α1-adrenergic receptor activation. This effect was not observed in the absence of the inhibitor. These data suggest that although phenylephrine likely activates both α and β-adrenergic receptors in the uterus, the β adrenoceptor activation effect plays a dominant role. Thus, it appears that there may be a complex interplay between these two signalling pathways in the uterine smooth muscles.

Acting via the β2-adrenoceptor, phenylephrine activates adenylyl cyclase, thereby increasing the intracellular concentration of cAMP (Figs 4 and 7). cAMP is known to relax uterine smooth muscles; and it has been proposed that the cAMP-induced relaxation involves the activation of cAMP-dependent protein kinase A (PKA), which phosphorylates myosin light chain kinase (MYLK)^22^ and phospholipase C (PLC)^23^, inhibiting their activity. In the present study, we indeed found that phenylephrine not only increased the level of cAMP, but also inhibited the oxytocin-induced Ca^2+^ signalling in primary cultured mouse uterine smooth muscle cells, likely due to the cAMP-dependent inhibition of PLC. However, the role of PKA in the cAMP downstream signalling pathway remains unclear in the myometrium. It has been recently reported that the exchange protein directly activated by cAMP (EPAC) rather than PKA may play the major role in cAMP-induced uterine relaxation^24^. Further studies will be required to better understand the mechanism of cAMP-induced uterine relaxations.

Along with phenylephrine, we investigated the uterine effects of another widely used FDA approved drug, caffeine. We established that 2–10 mM caffeine inhibited mouse uterine contractions. In vascular SMCs, caffeine was reported to induce a small initial contraction followed by a relaxation. The transient caffeine-induced contraction is due to the activation of ryanodine receptors on the sarcoplasmic reticulum^25^. In our case, we only observe caffeine-induced relaxations in the uterus. It is consistent with a recent report that none of the RyR subtypes (RyR1-3) plays a role in the regulation of contractions in mouse myometrial SMCs^26^.

The relaxant effect of caffeine is likely due to either its direct action on the contractile machinery of uterine smooth muscle cells or inhibition of the phosphodiesterase enzyme, which is important for modulating intracellular cAMP levels. Caffeine’s relaxant effect may also be due to the inhibition of the MLC kinase and decreasing actin filament binding to the phosphorylated myosin on the actin-myosin interaction^27,28^. However, our data presented in this paper indicate that the inhibition of smooth muscle phosphodiesterase leading to decreased intracellular cAMP degradation likely underlies the caffeine-induced relaxation of the mouse uterus.

We determined in this study that the EC_50_ value for the caffeine relaxant effect on the uterus was about 0.3 mM. In the victims of acute caffeine overdose, caffeine concentration may reach the range from 0.2 to 2 mM^29^. However, a cup of coffee contains only 80–175 mg of caffeine. Thus, after consuming one cup of coffee, a healthy volunteer’s blood plasma caffeine concentration should not exceed 0.01–0.05 mM. Therefore, moderate caffeine consumption (less than 200 mg per one sitting) is unlikely to affect uterine contractility.

In healthy non-pregnant women, transvaginal ultrasound and magnetic resonance imaging studies revealed the existence of endometrial wavelike movements, also known as uterine peristalsis^30–32^. Endometrial wavelike movements are driven by coordinated contractions of myometrial smooth muscles. The direction, intensity, and frequency of the endometrial wavelike movements change throughout the menstrual cycle^32,33^, with cervix-to-fundus uterine contractions dominating during the periovulatory phases. Cervix-to-fundus endometrial wavelike movements were also observed in the midfollicular phase, the late follicular phase, and the early luteal phase. Fundus-to-cervix endometrial wavelike movements were present in the follicular phase, specifically during the earlier follicular phase, assisting menstrual discharge; and opposing waves were observed up to five days after ovulation^30,32,33^. Endometrial wavelike movements may be important to intrauterine sperm transport toward the Fallopian tubes and for restricting the embryo implantation to the upper uterine segments^32^. *In vitro* fertilization studies indicate that impaired endometrial wavelike movements may lead to infertility, ectopic pregnancies, miscarriages, and endometriosis^33–35^. In this study, we observed that 1 μM phenylephrine almost completely inhibited spontaneous uterine wavelike contractions in mouse *ex vivo* female mouse reproductive tract preparations (Fig. 8g,h; Supplementary video #1, N = 5), suggesting that phenylephrine may potentially interfere with fertility. Consistently, mice transiently treated with a β2-adrenoceptor agonist at the time of embryo implantation exhibited the abnormal embryo spacing and increased midterm pregnancy loss^17^.

In the present paper, we provide evidence that phenylephrine exhibits strong reversible relaxant action in non-pregnant mouse uterus by triggering the cAMP signalling machinery, but larger doses of phenylephrine are needed to cause 50% inhibition in uterine rings from pregnant mice. Caffeine exerts relaxant actions only at non-physiologically high concentrations. Thus, our data suggest that it appears safe to consume moderate amounts of caffeinated drinks by healthy pregnant women during labour. But, it is advisable to limit the usage of over-the-counter phenylephrine-containing cold/flu remedies during term labour. Phenylephrine-containing medications may also need to be avoided in women trying to become pregnant.

## Materials and Methods

### Animals

All of the animal experiments were approved by the Indiana University School of Medicine Institutional Animal Care and Use Committee and strictly adhered to the guidelines described in the Guide for the Care and Use of Laboratory Animals published by the United States National Institutes of Health. 2–4 month old C57BL/6 mice were used in the study. The sexually mature mice were anesthetized using brief inhalation of isoflurane and euthanized by decapitation. The oestrous cycle stage was determined by examining cellular content from a vaginal douche as described elsewhere^36^. The uteri were isolated, cleaned from the surrounding connective tissue and cut into 3 mm length rings from the middle sections of the uterine horns (inset of Fig. 1a). Some uterine horns were cut-open longitudinally and used as tissue strips in the isometric tension experiments. Before the isometric tension recordings, the rings and strips were allowed to equilibrate for at least 1–2 hours in the oxygenated Krebs solution at 37 °C after multiple washes with the Krebs solution. In a subset of experiments, the mice were anesthetized by inhalation of 5% CO_2_ followed by euthanasia with decapitation to demonstrate that isoflurane did not affect the outcome of the phenylephrine experiments.

### Isometric tension experiments

The uterine rings were mounted on the wires of a wire myograph and lowered into 6 ml tissue baths that were filled with a standard Krebs buffer maintained at 37 °C. The buffer was continuously oxygenated with a gas mixture of 95% O_2_ and 5% CO_2_. The optimum length was determined as described elsewhere^37–40^. All drugs were added directly to the tissue baths while the contraction force was recorded.

### *Ex vivo* mouse uterine peristalsis assay

The entire intact female mouse reproductive tract, including the oviducts, uterine horns, uterine body, cervix, and vagina, was isolated and placed in a Petri dish filled with the oxygenated Krebs buffer. The tissue preparation was maintained at room temperature. The uterine peristalsis in the absence or presence of 1 μM phenylephrine was documented by digital video recording in MP4 video format using a Cannon EOS Rebel T6i camera.

### Cell culture

The isolated uterine horns were cut open longitudinally and were washed using DPBS. The endometrial layer was removed using microscissors and discarded, whereas the myometrium was minced. The tissue pieces of myometrium were dissociated in Hank’s Balanced Salt medium containing collagenase P (1 mg/mL, from Clostridium histolyticum, Roche, Indianapolis, IN, USA), 0.2 mM CaCl_2_, 0.125% bovine serum albumin (BSA) for 40 min, 37 °C. After collagenase treatment, the digested tissue pieces were gently triturated to disperse single smooth muscle cells. The obtained cells were washed with DPBS (3×) and then plated on glass coverslips. The cells were cultured in Eagle’s Minimum Essential Medium (EMEM, cat. No. 30-2003, ATCC, Manassas, VA, USA) supplemented with 10% fetal bovine serum, 100 U/mL penicillin, and 100 U/mL streptomycin for 24–48 h in the 5% CO_2_ incubator at 37 °C before the experiments. The human uterine smooth muscle cells (HUSMCs) were obtained from Lonza (CLONETICS Uterine Smooth Muscle Cell Systems, CC-2562) and cultured in the Lonza-recommended medium SmBM (Lonza,catlog No: cc-3181) supplemented with SmGM-2 SingleQuots (cat. No: cc-4149) in the 5% CO_2_ incubator at 37 °C.

### Solutions

The standard Krebs buffer contained (in mM): 130 NaCl, 5 KCl, 2 CaCl_2_, 1.2 NaH_2_PO_4_, 0.56 MgCl_2_, 25 NaHCO_3_, and 5 glucose. The 70 mM KCl solution contained (in mM): 65 NaCl, 70 KCl, 2 CaCl_2_, 1.2 NaH_2_PO_4_, 0.56 MgCl_2_, 25 NaHCO_3_, and 5 glucose. The extracellular solution for fluorescence imaging contained (in mM): 145 NaCl, 2.5 KCl, 2. CaCl_2_, 1 MgCl_2_, 10 HEPES, and 5.5 glucose (pH 7.2 adjusted with NaOH).

### cAMP reporter assay in live cells

The cyclic AMP (cAMP) reporter assay was purchased from Montana Molecular (Bozeman, MT, USA) and was performed as recommended by the manufacturer. The isolated M/HUSMCs were plated and grown for 24 h in culture medium supplemented with 10% fetal bovine serum or SmBM supplemented with SmGM-2 SingleQuots. The transduction reaction (25 µl Green Downward cADDis cAMP sensor, 20 µl Red Upward cADDis cAMP sensor and 4 µl 500 mM Sodium Butyrate per 1 ml complete medium) were added to the cells. The cells were incubated under normal growth conditions (5% CO_2_ and 37 °C) while being protected from light for 6 h. Then, the transduction solution was removed and 2 ml complete medium with sodium butyrate (2 mM) was added. Cells were incubated for an additional period of 24 h. Before measuring fluorescence, the culture medium was replaced with DPBS.

### Fluorescence imaging

The cultured uterine smooth muscle cells were incubated in Ca^2+^/Mg^2+^-containing PBS, supplemented with 4 μM Fura-2AM for an hour, and then incubated for another 30 min in PBS containing no Fura-2AM. A Till-Photonics single-cell fluorescence imaging system was used to monitor intracellular Ca^2+^ changes in isolated Fura-2-loaded M/HUSMCs. The fluorescence data are presented after subtracting the background fluorescence. The cultured cells were continuously superfused with the test solutions at a rate of 1.5 mL/min. The cells were depolarized with high external potassium solution (140 mM KCl, Fig. 4g) and only those cells that showed calcium increases in high potassium solution were used for analysis.

### siRNA assay

Silencer®Select siRNA against the β2-adrenoceptor (siRNA-Adrb2) and Silencer®Select Negative Control No. 1 siRNA were purchased from Ambion (Life Technology Corporation, Carlsbad, CA, USA; Cat. No. s62080 and 4390843, respectively). 25 pmol of β2-adrenoceptor or Negative Control siRNAs were added to the separate suspensions of freshly isolated mouse smooth muscle cells (~1 million cells in 100 μl AoSMC Nucleofector® Solution). The cell suspension combined with siRNA was transferred in a Nucleofector cuvette, which was then inserted into the Cuvette Holder of Amaxa’s Nucleofector II Device and Nucleofector Program U-025 was applied. The electroporated cells were added to 500 µl of EMEM supplemented with 10% fetal bovine serum and plated on glass coverslips. About 12 h later, the cells were transduced with the cAMP reporter plasmids and were cultured as described above for an additional 48 h period before beginning the fluorescence imaging experiments.

### Statistical analyses

The data were presented as mean ± standard error (S.E.) and analysed by the Sigma Plot12. The unpaired *t* test or Mann-Whitney Rank Sum Test was used to determine the significant different between two groups data with normally distributed populations and equal variances or non-normally distributed populations with different variances. When the two data sets were obtained from before and after application experiments with or without normally distributed populations with equal variances, the paired *t* test or Wilcoxon Signed Rank Test was used to determine whether there is a statistically significant difference. The one-way ANOVA test or the Kruskal-Wallis ANOVA on Ranks test followed by a post hoc all pairwise multiple comparison test was used to determine whether there is a statistically significant difference between multiple groups presenting normally distributed populations with equal variances or not. The Two Way ANOVA test followed by the Student-Newman-Keuls post hoc all pairwise multiple comparison test was uses to compare the experimental groups affected by two different factors when the data sets were normally distributed populations with equal variances. The data sets were considered significantly different if the *p* value was < 0.05. Throughout the paper, the “n” values indicate the number of tested cells; and the values of “N” are the number of used mice. During the analysis of the data obtained with cell cultures, the smooth muscle cells were functionally identified using applications of 140 mM KCl (Fig. 4g, inset). Only smooth muscle cells express voltage-gated calcium channels and show calcium influx upon depolarization with the 140 mM KCl solution. The total cell number represents a sum of all cells tested during three to six repeats of independent experiments performed on different days.

### Chemicals

All inorganic salts, phenylephrine, oxytocin, BSA, and DPBS were purchased from Sigma-Aldrich (St. Louis, MO, USA) and were of the highest purity. Penicillin and streptomycin were purchased from GIBCO-ThermoFisher Scientific (Gaithersburg, MD, USA). Prazosin and ICI118551 were purchased from Cayman Chemical (Ann Arbor, MI, USA).

## Electronic supplementary material

Supplementary Video 1

Supplementary Information
