# Supplementary material for: Phenylephrine, a common cold remedy active ingredient, suppresses uterine contractions through cAMP signalling
Source: Sci Rep. 2018 Aug 3;8:11666. doi: 10.1038/s41598-018-30094-5 (PMC6076280; doi:10.1038/s41598-018-30094-5)
Supplement: Supplementary file 2 — Supplementary Information [file 41598_2018_30094_MOESM2_ESM.pdf]

Phenylephrine, a common cold remedy active ingredient, suppresses uterine contractions through cAMP signalling

Xingjuan Chen, Marya Meroueh, Gabriela Mazur, Evan Rouse, Karmjot Singh, Christopher W. Stamatkin, and Alexander G. Obukhov

Supplementary Information

Supplementary video 1

Two entire female mouse reproductive tracts, including the oviducts, uterine horns, uterine body, cervix, and vagina, are shown in the video (see Fig. 8g for details). The tissue preparations were first placed in the standard oxygenated Krebs solution. The tissue preparations were manually moved to another Petri Dish containing the oxygenated Krebs solution supplemented with 1 μM phenylephrine. At the end of the movie, the reproductive tracts were returned to the Petri Dish containing the standard oxygenated Krebs solution. Phenylephrine markedly reduced spontaneous uterine peristalsis in the *ex vivo* female mouse reproductive tract preparations. Similar observations were made in three other mouse uteri (overall N=5). The experiment was performed at 23-25 °C. The uterine peristalsis in the absence or presence of 1 μM phenylephrine was documented by digital video recording in MP4 video format using a Cannon EOS Rebel T6i camera. The original MP4 video file was converted to a MOV video format file using Adobe Premier Pro software.
